# Supplementary material for: Hot spot prediction in protein-protein interactions by an ensemble system
Source: BMC Syst Biol. 2018 Dec 31;12(Suppl 9):132. doi: 10.1186/s12918-018-0665-8 (PMC6311905; doi:10.1186/s12918-018-0665-8)
Supplement: Supplementary file 2 — All relevant MATLAB code. A simple Matlab implement of our predictor. (ZIP 19,742 kb) [file 12918_2018_665_MOESM2_ESM.zip › Additional file 2/Read ASA/ASA website/dbMPIKT/1U0SY.html]

 NetSurfP - Protein Surface Accessibility and Secondary Structure Predictions

  

|  |  |  |
| --- | --- | --- |
|  |  | NetSurfP - Protein Surface Accessibility and Secondary Structure Predictions  Technical University of Denmark |

  


---

```
# For publication of results, please cite:
# A generic method for assignment of reliability scores applied to solvent accessibility predictions.
# Bent Petersen, Thomas Nordahl Petersen, Pernille Andersen, Morten Nielsen and Claus Lundegaard
# BMC Structural Biology 2009, 9:51 doi:10.1186/1472-6807-9-51
#
# Column 1: Class assignment - B for buried or E for Exposed - Threshold: 25% exposure, but not based on RSA
# Column 2: Amino acid
# Column 3: Sequence name
# Column 4: Amino acid number
# Column 5: Relative Surface Accessibility - RSA
# Column 6: Absolute Surface Accessibility
# Column 7: Z-fit score for RSA prediction
# Column 8: Probability for Alpha-Helix
# Column 9: Probability for Beta-strand
# Column 10: Probability for Coil
E G  1U0SY                  1    0.867  68.257   0.767   0.003   0.003   0.994
E K  1U0SY                  2    0.273  56.136   1.338   0.004   0.514   0.481
B R  1U0SY                  3    0.226  51.800   1.811   0.011   0.918   0.071
B V  1U0SY                  4    0.033   5.026   0.771   0.006   0.962   0.032
B L  1U0SY                  5    0.038   6.903   0.740   0.001   0.959   0.040
B I  1U0SY                  6    0.039   7.252   0.891   0.001   0.959   0.040
B V  1U0SY                  7    0.030   4.534   0.978   0.001   0.900   0.099
B D  1U0SY                  8    0.205  29.584   0.863   0.005   0.336   0.660
E D  1U0SY                  9    0.263  37.855   0.013   0.018   0.019   0.964
B A  1U0SY                 10    0.231  25.489   0.749   0.622   0.015   0.363
E A  1U0SY                 11    0.485  53.491   1.392   0.923   0.002   0.076
B F  1U0SY                 12    0.207  41.505   1.185   0.923   0.002   0.076
B M  1U0SY                 13    0.026   5.123   1.299   0.970   0.001   0.030
B R  1U0SY                 14    0.162  37.052   0.309   0.970   0.001   0.030
E M  1U0SY                 15    0.360  72.056   1.654   0.970   0.001   0.030
B M  1U0SY                 16    0.091  18.129   0.570   0.970   0.001   0.030
B L  1U0SY                 17    0.018   3.241   1.575   0.970   0.001   0.030
B K  1U0SY                 18    0.242  49.841   1.366   0.970   0.001   0.030
E D  1U0SY                 19    0.353  50.867   1.376   0.970   0.001   0.030
B I  1U0SY                 20    0.080  14.744   1.378   0.923   0.002   0.076
B I  1U0SY                 21    0.039   7.270  -0.005   0.858   0.002   0.139
E T  1U0SY                 22    0.561  77.769   0.659   0.600   0.003   0.397
E K  1U0SY                 23    0.474  97.564   0.695   0.321   0.003   0.675
E A  1U0SY                 24    0.633  69.724   0.488   0.016   0.005   0.979
E G  1U0SY                 25    0.659  51.855   0.317   0.003   0.003   0.994
B Y  1U0SY                 26    0.056  11.967   0.921   0.004   0.085   0.910
E E  1U0SY                 27    0.439  76.711   1.699   0.002   0.816   0.182
B V  1U0SY                 28    0.065   9.929   1.442   0.001   0.959   0.040
B A  1U0SY                 29    0.090   9.918   0.973   0.001   0.959   0.040
B G  1U0SY                 30    0.081   6.375   0.677   0.001   0.959   0.040
B E  1U0SY                 31    0.176  30.782   0.524   0.001   0.900   0.099
B A  1U0SY                 32    0.027   3.030   0.215   0.004   0.514   0.481
E T  1U0SY                 33    0.440  61.042   0.119   0.018   0.047   0.935
E N  1U0SY                 34    0.253  37.068   0.692   0.018   0.019   0.964
B G  1U0SY                 35    0.031   2.479  -0.513   0.923   0.002   0.076
E R  1U0SY                 36    0.423  96.913   1.785   0.970   0.001   0.030
E E  1U0SY                 37    0.375  65.530   1.802   0.970   0.001   0.030
B A  1U0SY                 38    0.014   1.532   1.590   0.970   0.001   0.030
B V  1U0SY                 39    0.063   9.729   1.119   0.970   0.001   0.030
E E  1U0SY                 40    0.501  87.490   2.040   0.970   0.001   0.030
B K  1U0SY                 41    0.147  30.176   1.699   0.970   0.001   0.030
B Y  1U0SY                 42    0.027   5.791   0.718   0.970   0.001   0.030
E K  1U0SY                 43    0.487 100.094   1.143   0.923   0.002   0.076
E E  1U0SY                 44    0.723 126.291   1.027   0.782   0.003   0.216
E L  1U0SY                 45    0.242  44.383   1.139   0.321   0.003   0.675
E K  1U0SY                 46    0.502 103.220   0.858   0.016   0.005   0.979
B P  1U0SY                 47    0.066   9.337   0.569   0.005   0.015   0.979
B D  1U0SY                 48    0.206  29.713   0.623   0.004   0.085   0.910
B I  1U0SY                 49    0.022   4.144   1.407   0.001   0.900   0.099
B V  1U0SY                 50    0.023   3.612   1.217   0.001   0.959   0.040
B T  1U0SY                 51    0.030   4.203   0.939   0.001   0.959   0.040
B M  1U0SY                 52    0.024   4.842   1.285   0.001   0.959   0.040
B D  1U0SY                 53    0.046   6.701  -0.172   0.002   0.816   0.182
B I  1U0SY                 54    0.061  11.303  -0.110   0.004   0.420   0.576
E T  1U0SY                 55    0.271  37.643  -0.443   0.021   0.279   0.699
B M  1U0SY                 56    0.059  11.866   1.005   0.018   0.088   0.893
B P  1U0SY                 57    0.222  31.559   0.256   0.005   0.015   0.979
E E  1U0SY                 58    0.616 107.545  -1.146   0.005   0.015   0.979
B M  1U0SY                 59    0.202  40.420  -0.209   0.018   0.019   0.964
E N  1U0SY                 60    0.320  46.775  -0.803   0.053   0.005   0.942
B G  1U0SY                 61    0.034   2.684  -1.005   0.858   0.002   0.139
B I  1U0SY                 62    0.124  22.940   0.860   0.970   0.001   0.030
E D  1U0SY                 63    0.378  54.412   1.125   0.970   0.001   0.030
B A  1U0SY                 64    0.029   3.174   0.781   0.970   0.001   0.030
B I  1U0SY                 65    0.020   3.681   1.571   0.970   0.001   0.030
E K  1U0SY                 66    0.331  68.046   1.938   0.970   0.001   0.030
E E  1U0SY                 67    0.346  60.376   2.457   0.970   0.001   0.030
B I  1U0SY                 68    0.019   3.570   1.494   0.970   0.001   0.030
B M  1U0SY                 69    0.230  46.023   1.608   0.923   0.002   0.076
E K  1U0SY                 70    0.726 149.276   1.144   0.858   0.002   0.139
E I  1U0SY                 71    0.510  94.350   0.880   0.600   0.003   0.397
B D  1U0SY                 72    0.256  36.918  -0.461   0.053   0.005   0.942
E P  1U0SY                 73    0.549  77.903  -0.506   0.016   0.005   0.979
E N  1U0SY                 74    0.705 103.271   1.482   0.005   0.015   0.979
B A  1U0SY                 75    0.031   3.460   0.831   0.005   0.045   0.951
B K  1U0SY                 76    0.175  36.018   1.528   0.004   0.420   0.576
B I  1U0SY                 77    0.020   3.756   1.375   0.001   0.959   0.040
B I  1U0SY                 78    0.018   3.367   1.570   0.000   0.983   0.017
B V  1U0SY                 79    0.018   2.690   1.514   0.000   0.983   0.017
B C  1U0SY                 80    0.018   2.555   1.522   0.001   0.959   0.040
B S  1U0SY                 81    0.027   3.211   0.564   0.002   0.816   0.182
B A  1U0SY                 82    0.040   4.430  -0.684   0.005   0.336   0.660
B M  1U0SY                 83    0.242  48.424   0.666   0.004   0.085   0.910
E G  1U0SY                 84    0.364  28.655  -0.593   0.005   0.015   0.979
E Q  1U0SY                 85    0.383  68.368  -0.562   0.058   0.017   0.925
E Q  1U0SY                 86    0.362  64.707  -0.493   0.858   0.002   0.139
E A  1U0SY                 87    0.515  56.775   0.707   0.923   0.002   0.076
B M  1U0SY                 88    0.167  33.497   1.339   0.923   0.002   0.076
B V  1U0SY                 89    0.037   5.672   0.583   0.970   0.001   0.030
B I  1U0SY                 90    0.197  36.501   1.275   0.970   0.001   0.030
E E  1U0SY                 91    0.342  59.817   1.851   0.970   0.001   0.030
B A  1U0SY                 92    0.021   2.347   0.241   0.970   0.001   0.030
B I  1U0SY                 93    0.087  16.151   0.630   0.923   0.002   0.076
E K  1U0SY                 94    0.520 106.943   1.355   0.858   0.002   0.139
B A  1U0SY                 95    0.158  17.368   0.378   0.502   0.002   0.495
B G  1U0SY                 96    0.178  14.016  -0.252   0.176   0.004   0.820
B A  1U0SY                 97    0.040   4.386  -1.163   0.058   0.017   0.925
E K  1U0SY                 98    0.410  84.275  -0.249   0.053   0.043   0.903
B D  1U0SY                 99    0.233  33.503  -1.262   0.056   0.142   0.802
B F  1U0SY                100    0.140  28.178   0.501   0.066   0.296   0.638
B I  1U0SY                101    0.086  15.855   0.531   0.066   0.296   0.638
B V  1U0SY                102    0.232  35.674   0.529   0.019   0.141   0.840
B K  1U0SY                103    0.273  56.136   0.848   0.005   0.045   0.951
E P  1U0SY                104    0.600  85.083  -0.106   0.005   0.045   0.951
B F  1U0SY                105    0.116  23.221  -0.149   0.005   0.015   0.979
E Q  1U0SY                106    0.540  96.408   1.559   0.005   0.015   0.979
B P  1U0SY                107    0.226  32.112  -0.237   0.923   0.002   0.076
E S  1U0SY                108    0.669  78.372   1.242   0.970   0.001   0.030
E R  1U0SY                109    0.375  85.967   1.814   0.970   0.001   0.030
B V  1U0SY                110    0.028   4.273   0.929   0.988   0.000   0.012
B V  1U0SY                111    0.159  24.469   1.579   0.988   0.000   0.012
E E  1U0SY                112    0.505  88.154   1.605   0.988   0.000   0.012
B A  1U0SY                113    0.157  17.268   1.527   0.970   0.001   0.030
B L  1U0SY                114    0.041   7.470   0.035   0.975   0.003   0.022
E N  1U0SY                115    0.454  66.422   1.887   0.938   0.007   0.055
E K  1U0SY                116    0.562 115.562   1.922   0.858   0.002   0.139
B V  1U0SY                117    0.162  24.946   0.718   0.694   0.003   0.303
E S  1U0SY                118    0.734  86.072  -0.644   0.016   0.005   0.979

Explain the output.  Go back.
```

---
